# Diagnosis of West Nile Virus Human Infections: Overview and Proposal of Diagnostic Protocols Considering the Results of External Quality Assessment Studies

**DOI:** 10.3390/v5102329

**Published:** 2013-09-25

**Authors:** Vittorio Sambri, Maria R. Capobianchi, Francesca Cavrini, Rémi Charrel, Olivier Donoso-Mantke, Camille Escadafal, Leticia Franco, Paolo Gaibani, Ernest A. Gould, Matthias Niedrig, Anna Papa, Anna Pierro, Giada Rossini, Andrea Sanchini, Antonio Tenorio, Stefania Varani, Ana Vázquez, Caterina Vocale, Herve Zeller

**Affiliations:** 1 Operative Unit of Clinical Microbiology, Regional Reference Centre for Microbiological Emergencies, S. Orsola-Malpighi University Hospital, Bologna 40138, Italy; E-Mails: vittorio.sambri@unibo.it (V.S.); cavrini@aosp.bo.it (F.C.); paolo.gaibani@unibo.it (P.G.); anna.pierro@unibo.it (A.P.); giada.rossini@unibo.it (G.R.); stefania.varani@unibo.it (S.V.); caterina.vocale@unibo.it (C.V.); 2 National Institute for Infectious Diseases (INMI) “L. Spallanzani”, Rome 00149, Italy; E-Mail: capobianchi@inmi.it; 3 UMR_D 190 “Emergence des Pathologies Virales”, APHM Public Hospitals of Marseille, EHESP French School of Public Health & IHU Mediterranee Infection, IRD French Institute of Research for Development, Aix Marseille University, 13005, Marseille, France; E-Mail: remi.charrel@univ-amu.fr (R.C.); 4 Centre for Biological Threats and Special Pathogens (ZBS-1), Robert Koch-Institut, Berlin 13353, Germany; E-Mails: donosoo@rki.de (O.D.-M.); escadafal@rki.de (C.E.); NiedrigM@rki.de (M.N.); sanchini@rki.de (A.S.); 5 National Microbiology Centre, Instituto de Salud Carlos III, Madrid 28220, Spain; E-Mails: francolet@isciii.es (L.F.); atenorio@isciii.es (A.T.); a.vazquez@isciii.es (A.V.); 6 NERC Centre for Ecology and Hydrology, Wallingford, Oxon OX10 8BB, UK; E-Mail: eag@ceh.ac.uk (E.A.G.); 7 Department of Microbiology, Medical School, Aristotle University of Thessaloniki, Thessaloniki 54124, Greece; E-Mail: annap@med.auth.gr; 8 European Public Health Microbiology Training Programme (EUPHEM), European Centre for Disease Prevention and Control, Stockholm 171 83, Sweden; 9 European Centre for Disease Prevention and Control, Stockholm 171 83, Sweden; E-Mail: herve.zeller@ecdc.europa.eu

**Keywords:** West Nile virus, state of the art laboratory diagnostic, external quality assurance

## Abstract

West Nile virus, genus *Flavivirus*, is transmitted between birds and occasionally other animals by ornithophilic mosquitoes. This virus also infects humans causing asymptomatic infections in about 85% of cases and <1% of clinical cases progress to severe neuroinvasive disease. The virus also presents a threat since most infections remain unapparent. However, the virus contained in blood and organs from asymptomatically infected donors can be transmitted to recipients of these infectious tissues. This paper reviews the presently available methods to achieve the laboratory diagnosis of West Nile virus infections in humans, discussing the most prominent advantages and disadvantages of each in light of the results obtained during four different External Quality Assessment studies carried out by the European Network for ‘Imported’ Viral Diseases (ENIVD).

## 1. Introduction

West Nile virus (WNV) is an enveloped spherical, single-stranded RNA flavivirus that is transmitted to birds by ornithophilic mosquitoes, mainly belonging to the genus *Culex* [1] and is occasionally transmitted to mammalian hosts [2]. The geographical area of WNV circulation encompasses most of Africa, Israel, North America and South America, Australia and scattered areas in southern and central Europe, including Russia, Czech Republic, Hungary, Greece, Romania, Italy, Southern France, Portugal, Turkey and Spain [2,3,4]. At least two distinct genetic lineages have been identified among the WNV isolates in diverse areas [1]: lineage 1 includes most of the American, European and some African strains, whereas lineage 2 contains mainly sub-Saharan African isolates, although some lineage 2 strains have been detected in humans and mosquitoes outside Africa [5,6,7,8]. Additional lineages have been proposed: lineages 3, 4 and 5 include viruses isolated from Czech Republic (Rabensburg strain), Caucasus and India, respectively [9,10,11]. A sixth lineage was recently described in Indonesia and a putative seventh lineage has been identified in Spain [11].The strains belonging to lineages 1 and 2 have up to 30% nucleotide divergence [12]. This wide diversity together with the elevated threat for human health posed by WNV infections resulted in the development of a variety of methods for laboratory diagnosis [13]. It is important to emphasise that about 80% of the human infections caused by WNV remain asymptomatic and therefore approximately 20% of cases become clinically evident [2]. The clinical syndromes associated with WNV human infections are predominantly mild flu-like fevers (WNV fever) of which less than 1% develops severe neuroinvasive disease [14]. For detail about the case classification of the WNV infection and for proposals concerning laboratory diagnosis of this virus please refer to the conclusion of this paper.

This paper reviews the currently available techniques for the identification of WNV infection in humans. Four External Quality Assessment (EQA) studies have been performed: two for the molecular diagnostics of WNV infections, and two for the serological methods. The results of these studies are presented at the end of this review, focusing on the main diagnostic problems. 

Currently, the laboratory methods for the diagnosis of infection by WNV belong to two main categories: serology and viral detection.

## 2. Virus Detection

***A.*** ***Virus isolation***. WNV is not readily isolatable from tissues, plasma, serum and CSF samples in cell culture using either mammalian or mosquito derived cell lines, such as Vero E6, RK-13, AP61 or C6/36 [15,16,17,18]. Moreover, these procedures must be performed under biosafety level 3 conditions [1] and it generally takes up 7 to days before the appearance of CPE. Virus isolation is most readily accomplished if the following procedures for collecting the samples are adhered to; whenever possible, the samples should be collected under sterile conditions, in collecting media containing antibiotics and immediately refrigerated at 4 °C. Under optimal conditions the samples should be inoculated onto susceptible cells in culture within minutes of collection but when this is not possible, the time interval should be kept to a minimum and always less than 24 hours. In general these conditions can be met in reference laboratories and dedicated research facilities [13]. The isolation of WNV strains provides the added value of allowing further studies and research on pathogenesis, genetic variation evolution, epidemiology, *etc.*

***B. Molecular methods***. WNV genomes in peripheral blood are usually detectable from 2–3 days to 14–18 days post-infection [19]. During this time it is usually possible to identify the viral RNA in serum and/or plasma samples. In addition the detection of viruria by molecular methods has been demonstrated to be a useful tool that enables the detection of WNV genomes, even after prolonged times post-infection [5,20]. For the routine detection of WNV RNA using molecular techniques there are two distinct diagnostic settings: the first involves blood and organ donation screening from subjects living in an area where WNV circulation is known to be occurring, and the second involves the identification of viral genomes in serum, plasma and CSF samples from patients presenting with a clinical picture typical of WNV infection [21]. 

***Blood and organ safety screening***. Transmission of WNV between humans during blood transfusion and tissue and or organ transplantation has been recorded in the USA [22] and recently in Europe [23,24,25,26]. In order to increase the biological safety of blood and organ donations, a screening policy has been implemented [27,28] in areas where human cases of WNV related diseases are known to be present, since it is believed that at least 80% of the WNV infected population remains asymptomatic during the phase in which viral RNA is actually detectable in blood [3]. In this diagnostic setting, the basic requirement for laboratories is to achieve very high sensitivity values, since these techniques must be applied in a healthy and totally asymptomatic population where the hypothetic WNV load could be as low as 100 genome copies/mL. Two commercial nucleic acid amplification tests (NATs) are presently able to detect genomes at these low levels. The first is based on polymerase chain reaction (PCR) technology [29] and is manufactured by Roche Diagnostics (Mannheim, Germany) and the second is a transcription mediated amplification (TMA) test [30] available from Novartis Diagnostics (Emeryville, CA, USA). Both assays are patented and the target sequence is not available to users. They are fully automated on high throughput instruments and allow the testing of hundreds of plasma samples per day, with an elevated numerical performance as required by the blood bank workflow. The analytical sensitivity of these tests (as stated by the manufacturers) is 9.8 [Confidence Interval (CI) 95%: 6.5–27.3] and 40.3 (CI 95%: 35.1–47.8) genome equivalents/mL, for the TMA and PCR, evaluated on lineage 1 WNV strains, respectively. These sensitivity values are within the standard for WNV blood screening products that have been set by the US Food and Drug Administration within 100 copies/mL [31]. The specificity of these methods is considered to be quite high [32,33]. However, false positive reactions were reported due to the presence of Usutu (USUV), a virus closely related to WNV [34,35]. Depending on the local epidemiological prevalence of WNV infections, blood screening can be performed on individual samples (ID-NAT) or in pooled samples (usually 6 or 16 mini-pool specimens: MP-NAT), the ID-NAT test being the more sensitive of the two [36,37,38]. As an alternative to these techniques, the use of manually performed Real time RT-PCR (QIAGEN, Hilden, Germany) and nucleic acid sequence based amplification (NASBA) have been recently reported [39] in a population of tissue and organ donors, with good performance in terms of turnaround time and clinical performance. 

***Patients with suspected WNV neuroinvasive disease or WN fever****.* The viral load in biological specimens (blood, CSF, urine) from patients with suspected WNV infection is hypothesized to be higher than in asymptomatic infected subjects and consequently the diagnostic methods do not need the same high sensitivity that is required for the screening tests used for asymptomatic individuals.

***Reverse Transcription PCR (RT-PCR)***. The sensitivity of conventional RT-PCR for WNV detection depends mainly on the target sequence. A test based on primers that target the C gene (3') and the prM gene (5') detected viral RNA equivalent to approximately 0.1 plaque forming units (PFU) derived from a cell grown NY99 isolate [40]. Nested RT-PCR techniques can increase by up to 10 fold the the RNA detection threshold but these methods are more prone to contamination, require a longer time to perform and increased involvement of laboratory personnel [40]. The use of pan-specific RT‑PCR techniques, capable of identifying most of the flaviviruses and mainly based on nested assays [41,42], can potentially differentiate WNV related disease from other infections caused by flaviviruses. The amplicons obtained using these labour intensive methods need to be sequenced and the obtained electropherograms analysed in order to identify the infecting virus. No precise indication can be provided presently about methods that can be used to differentiate the diverse WNV lineages one from another. Most of the recently published papers in the field of WNV phylogeny are based on the analysis of the NS or E genes.

***Real-Time RT PCR***. These methods are generally rapid and reliable and can be used for detection of WNV on a large variety of samples, including non-human specimens, such as animal tissues and mosquitoes [43]. Different technical modifications have been proposed in the last 10 years. In general, the TaqMan probe-based assays are quite sensitive (approximate detection limit is 0.1 PFU of viral RNA) [44] but sometimes incapable of detecting emerging genomic variants of the virus [45]. The SYBR Green based tests are as sensitive as the TaqMan tests, but less specific [46]. The decreased specificity could be both a disadvantage, since false positive results can be generated by diverse viruses, and an advantage, given the broader spectrum of WNV variants that can be identified [46]. Another potential disadvantage of real-time RT-PCR is the represented by the generally short amplicons obtained, that are not suitable for sequencing and molecular characterization. Given the strain variation of WNV from season to season and in different geographical locations, new diagnostic molecular approaches have been proposed. A multiplex PCR-ligase detection reaction assay (RT‑PCR/LDR) based on the contemporary amplification of three different genomic regions (one in the coding sequence of NS2 and two in the nonstructural gene NS5) minimized the risk of false negative results due to WNV genetic variants [47]. Another method, based on genome-wide RT-PCR [45] overcame the undetectability of WNV isolates with genomic differences. As mentioned, WNV isolates have been phylogenetically grouped into 7 different lineages [11] and lineages 1 and 2 overlap in some areas of Europe [9]. In order to distinguish between lineage 1 and 2, a novel real-time quantitative RT-PCR test has been developed. This test showed an extremely high differentiation capacity on different cell derived WNV isolates at the level of 2–4 genome copies [48]. WNV overlaps geographically with several other arboviruses including flaviviruses, alphaviruses and some bunyaviruses and as the number of recognised emerging viruses increases in both tropical and sub‑tropical regions [49,50,51,52], new multiplex RT-PCR assays have been developed to facilitate simultaneous identification, for example, of chikungunya virus, DENV [53], JEV and WNV in patient samples [54]. As these techniques improve the early detection of multiple arboviruses is becoming a practical reality with the potential for rapid and cost-effective differential diagnosis and epidemiological surveillance.

***C. Immunohistochemistry***. The detection of WNV antigen using histochemical protocols in tissues obtained from fatal encephalitis cases has been available for diagnostic purposes for many years. In today’s laboratories this procedure is performed rarely and usually in order to improve the certainty of a clinical diagnosis in cases where laboratory data are minimal [55]. Data on the diagnostic performance of this assay have been mainly obtained in animals and the specificity of this method depends largely on the specificity and quality of the antiserum used. The sensitivity in many cases can be low and is often dependent on the amount of WNV present in the tissue and the quality of the tissue under investigation [56,57].

It is noteworthy to underline that most of the diagnostic approaches described in this paragraph could be hampered in the daily routine use for the laboratory diagnosis of WNV infection by the lack of standardized protocols and by the uncertainty about their specificity and sensitivity.

## 3. Serological Methods

Specific antibody detection still remains the most widely used approach for the diagnosis of WNV infection in humans. In order to understand the application of serology for WNV diagnosis, it is useful to remember that the mean times from the detection of viral RNA to IgM and IgG seroconversion are approximately 4 and 8 days respectively, as determined in a study performed amongst more than 200 WNV viraemic blood donors in the USA [19]. The main weakness that limits the clinical relevance of serological methods is the broad antigenic cross-reactivity that exists between all flaviviruses: the quite specific viral envelope (E) protein neutralising antibody response is often combined with less specific tests based on detection of antibodies against the membrane (M) and non-structural (NS) proteins of which the amino acid sequences are more conserved amongst the flaviviruses [13]. Based on this consideration, the principal serological methods can be subdivided into two main groups: the first includes the enzyme-linked immunosorbent assays (ELISAs) and immunofluorescence (IF) based tests; the second includes the Plaque Reduction Neutralization Test which can be carried out using a highly sensitive 50% or less-sensitive 90% endpoint (PRNT_50,_ and PRNT_90_ respectively), both of which require the constant availability of standardised-validated infectious viruses and appropriate cell cultures. The hemagglutination-inhibition test (HIA) is still used to detect pan-flavivirus immune response whereas the complement fixation test (CFT) is rarely used in today’s laboratories. The techniques included in the first group are widely used due to their relative applicability in routine laboratory and the ability to automate a part of the workflow but they are less specific as a consequence of their inability to distinguish between WNV-specific and cross-reactive antibody responses [13]. Thus, any positive result identified using these methods must be confirmed by the more specific tests, *i.e.*, those that constitute the second group. For serosurveys involving individuals that appear healthy but may have been infected sub-clinically, the gold-standard for detecting immune responses is the PRNT_50_ method which is able to detect, specifically, low titre, low avidity immune responses. The PRNT_90_ assay, by definition, is less sensitive than the PRNT_50_ assay and therefore is more appropriate for studies that involve the detection of immune responses in individuals that have presented with clinically apparent infection and have almost certainly developed detectable viraemia during their clinical infection. It is important to emphasise that in either of these analyses, the test should follow the guidelines of the World Health Organization [58] and should include control standardised viruses that are known to be readily neutralisable and to be antigenically closely related but distinct species from WNV. This second group of techniques, particularly PRNT assays are labour intensive and are generally limited to reference or dedicated research laboratories, where appropriate biosafety level could be attained [1].

***A. PRNT***. The flavivirus antibody response is directed against both family cross-reactive epitopes and virus specific epitopes [59]. The E protein is one of the most prominent immunogenic polypeptides and elicits most of the neutralizing antibodies (NAbs) [60]. This test is generally performed following standard protocols [61]. In detail, the method defined for Dengue by the WHO could be adapted for WNV [62] describes a standardised protocol for determining PRNT_90_ titres of sera from patients. The same protocol could be used also to determine a PRNT_50_ titre in sera obtained from sub-clinical infections. This last method is particularly useful to carry out a serosurvey on healthy individuals to determine whether or not they have previously experienced infection with WNV (or other viruses). Given the broad antigenic cross-reactivity between different flaviviruses and the diverse neutralizing activity of the human IgG subclasses [63], both PRNT_90_ and PRNT_50_ require evaluation of the NAbs against a panel of related viruses [13]. Based on the present known epidemiology of flavivirus human infections in Europe, the following viruses, grown either in mammalian cell lines e.g., Vero E6 or mosquito-derived cells such as C6/36, should be included for differential diagnostic purposes and to act as the controls for cross-reactivity reactions [64]. Two strains of WNV lineage 1: one recently isolated in Europe from human infections [15], preferably at a low passage number in cell culture, and one reference strain, for example, the Italia 1998 or France 2000; while the use of old strains such as Eg 101 should be avoided. One strain of WNV lineage 2, since the presence of this lineage has recently been identified in the Greek ongoing epidemic [6,65]. USUV which is now believed to be present in most of central Europe [12] and has recently been reported as causing human infections [66,67]. Under selected clinical and epidemiological circumstances, such as when testing samples obtained from patients that travelled in diverse geographical areas prior to be tested, other flaviviruses should be included in this panel, as follows. Japanese encephalitis virus (JEV) which is widespread in Asian countries [68] from where a large human migration stream to Europe is presently occurring and whose genome has recently been putatively identified in vectors in Italy [69,70,71]. Tick‑borne encephalitis virus (TBEV) that is widespread in central and eastern European countries [72]. In order to have a more complete panel, two additional viruses should also be included: one serotype of dengue virus (DENV) in light of the large number of imported cases within the European Union territory, [73,74,75,76] in travellers returning from tropical endemic areas and of the recently reported autochthonous activity of DENV in scattered areas in France [50] and Croatia [51]. Finally, yellow fever virus (YFV) should be included in this panel to ensure the absence of YFV-vaccine derived NAbs [77]. As a general rule, given the complicated pattern of antibody cross-reactivity among the family *Flaviviridae* [78,79], NAb titres should be consistently higher against WNV than the titres detected against the control viruses in the panel. There are also technical variations in the testing protocol and this issue must be considered when comparing PRNT results obtained in different laboratories. For example, variation to the techniques used for the detection of NAbs could include the incubation time (usually from 72 to 120 hours), the method for cytopathic effect (CPE) detection (direct microscopy, staining with a vital dye, detection by IF or by automated colorimetric detection [80]) and the diameter of the dish/well used to support the cell monolayer infected with the viruses (Micro Neutralisation Titre Assay: MNTA [81]. 

***B. HIA and CFT***. These tests, nowadays, are rarely used in the diagnosis of WNV infections in humans. The HIA test requires treatment of each serum to remove non-specific inhibitors [13]. It also requires the daily availability of fresh erythrocytes to maintain a high performance. Moreover, HIA is broadly cross-reactive. CFT is very labour intensive both in the analytical stage and in the reading phase, and the test specificity and sensitivity suffer from the lack of standardization of the antigenic preparations obtained from different sources [82]. All the tests reported above at points A and B are technically difficult, time consuming, relatively insensitive (with the possible exception of the PRNT50 test) during early infection and require several days before a result can be delivered. Consequently in the diagnostic laboratories of today, most serological diagnostic tests are performed by EIA and IF which are both technically simpler to perform. In addition, they can be highly specific when they incorporate monoclonal Abs (MAbs). 

***C. EIAs***. These methods can be subdivided into three main categories: (1) the IgM antibody-capture EIA (MAC-EIA), (2) the indirect IgG EIA and (3) the epitope blocking EIA. All of these methods have the advantage of rapidity and reproducibility when compared with those detecting NAbs and with HIA and CFT.

***MAC-EIA***. This test is suitable for routine detection of immune responses resulting from an acute viral infection. The IgM response is usually detectable within the first week of WNV infection, sometimes during the pre-symptomatic stage, in plasma, serum or cerebrospinal fluid (CSF) and generally persists for up to 47 days in plasma/serum with prolonged persistence in the CSF up to 100–199 days post infection [83] and up to 1 year in plasma/serum of viraemic blood donors [84]. The standard commercially available MAC-EIAs use plates coated with antibodies anti-human IgM and WNV antigens obtained from the brain of experimentally infected mice. As the secondary immunoglobulin, monoclonal antibodies (MAbs) directed to WNV conjugated with horse radish peroxidase can be used [85]. The serum dilution for screening is generally at 1:400. This test has a calculated sensitivity of 91.7% with a specificity of 99.2% [86]. Some technical modification of the IgM detection has recently been proposed in order to increase the sensitivity, specificity and clinical performance of the tests. In particular, the introduction of a ratio method in the calculation of the IgM EIA results has been demonstrated to eliminate nonspecific reactivity [87]. Further, the use of recombinant highly specific antigens, such as the epitopes located in the domain III of the E protein [88], the whole recombinant E protein produced in insect larvae [89], the Virus-Like Particles (VLPs) from preM and E proteins [90,91], the NS1, 3 and 5 proteins [92,93] have been proposed with promising results in order to increase the standardization of the antigen preparation and to reduce the costs of these assays. It is generally accepted that the identification of an IgM response is a sign of probable WNV infection that needs to be further confirmed by other laboratory criteria, primarily by a fourfold increase of the serum antibody (Ab) titres between different samples collected during the acute and convalescent stage of the disease. The presence of virus-specific IgM in the CSF is generally accepted as direct evidence of infection, thus prompting to identify the case as a confirmed one, according to the EU case definition [94,95]. Although in general results of MAC-EIAs correlate well with the PRNT_90_ and the cross-reactivity with IgM Abs resulting from infection by JEV serocomplex viruses is generally lower than for that of IgG antibody responses [79,85,95], false positive results have been reported with the routine use of MAC-EIA [96,97,98]. 

***Indirect IgG EIA***. The detection of WNV IgG by EIAs is now standardized and these methods are widely used for the determination of immune status to WNV either in suspected patients or in healthy asymptomatic populations [65,99,100,101,102,103]. A major limitation of these tests is, firstly, their restricted clinical specificity due to extensive cross-reactions with flaviviruses. For this reason it is recommended that the detection of IgG should be performed in combination with the MAC-EIA in all cases of suspected infection, since persistence of IgM may last for up to 6 weeks after disease onset [19,83,84]. All the cases of positive IgG detected by this technique need to be further evaluated and confirmed either by a PRNT_90_ test or by an increase of the IgG response later on in the course of infection. The reported specificity and sensitivity values for the most commonly used commercially available IgG EIA techniques are about 92%–93% and 97%–98%, respectively [104,105]. In order to estimate the time course of infection, two commercially available EIA methods (Focus Diagnostics, Cypress, CA, USA and Euroimmun, Lübeck, Germany) that evaluate the avidity of the IgG immune response during WNV symptomatic disease have been used. In sera obtained within the first 30 days after the onset of infection the IgG avidity was lower than 40%, whereas an avidity index higher than 55%–60% correlates with infections lasting at least 180 days [106,107]. The determination of IgG avidity is likely to provide additional useful data for clinical differentiation of recently and previously acquired WNV infections. A new test, based on the Immune complex (IC) ELISA method has recently been proposed for the detection of IgG, showing an elevated capability of discriminating between antibody response directed against WNV and TBEV [108]. Similar tests could be further developed in order to discriminate between WNV and USUV, JEV, Murray Valley encephalitis virus and Saint Louis encephalitis virus infections.

***Epitope blocking EIA***. A technical variant of the EIAs for the identification of IgG is the so called “Epitope-Blocking EIA”. This method is based on competition between the tested sera and WNV specific MAbs for binding to WNV antigens and was originally developed for diagnosis and prevalence studies in animals since it is species-independent [109]. This technique has also recently been evaluated for the diagnosis of human infection. The results obtained showed that this method is only applicable in populations not previously exposed to flaviviruses other than WNV [110] which results in a lack of correlation with the PRNT_90_ findings. Previous vaccination against JEV is another possible confounding factor for the interpretation of the results.

***D. IF***. The use of the IF assay in routine diagnosis of WNV infection is mainly dependent on the number of samples that need to be evaluated and the quality of training of laboratory personnel in interpreting the test results. Most of the tests used are developed “in-house” by individual laboratories usingWNV infected cell cultures. Consequently this technique suffers from a lack of standardization when results are compared between laboratories and would greatly benefit from an External Quality Assurance (EQA) evaluation as shown later. A commercially available IF test (Euroimmun, Germany) was shown to have about 94% specificity when compared with the PRNT_90_ test, in a study performed on 200 sera collected during a human WNV outbreak in South Africa [104]. In addition, new protocols for the IF testing have been developed for the detection of IgM and IgG antibodies. These methods have been reported to exhibit higher specificity than MAC EIA [111,112]. To reduce the non-specific reactivity (against related vector transmitted viruses or against cellular antigens) defined quantities of uninfected cells should be mixed with WNV-infected cells: the uninfected cells are obviously expected to remain non-reactive with tested sera.

In order to achieve practical recommendations about the possible routinely use of the serologic tests described above please refer to Table 1.

**Table 1:** The table below reports a proposal for the use of the laboratory tools described in this review.

Clinical Issue (biological sample)	Suggested laboratory tools
Diagnosis of patients with suspected WNV infection: neuroinvasive disease and WNV fever (serum and/or plasma, whole blood, CSF, urine).	It is advisable to prioritize the order of the tests as follows: MAC-EIA, Indirect IgG EIA, IF, virus isolation, RT-PCR, Real time RT-PCR, PRNT_90_
Evaluation of seroprevalence (serum and/or plasma)	PRNT_50_; Indirect IgG EIA, IF; Epitope blocking EIA
Screening of blood and organ donations (serum or plasma)	NATs [MAC-EIA and IF (IgG and IgM) are suggested to perform the serologic follow up of the NATs positive donors and as early additional testing in case of not confirmed NAT positivity]
Post-mortem evaluation (tissue biopsy)	Immunohistochemistry

## 4. External Quality Assurance Studies for the Serological and Molecular Diagnostics of WNV Infections

Four external quality assurance (EQA) studies assessing the quality of WNV diagnostics worldwide were performed or supported by the European Network for Diagnostics of ‘Imported’ Viral Diseases (ENIVD) [113]. A first study assessing the quality of molecular detection methods of WNV infections was conducted in 2006 [114] and a second one in 2011 [115]. Serological detection methods were assessed during a first EQA study in 2007 [105] and a second in 2012 [116]. 

For each EQA, participants were asked to analyse the provided EQA samples using the procedures routinely used by them in suspected human cases. Assay details, such as the type of method used, suppliers of commercial kits, protocols and references were requested. The participants could have the choice of the diagnostic procedure employed.

Regarding the first EQA of molecular detection methods, the overall diagnostic performance for WNV (2006) was disappointing compared with earlier EQA studies on emerging agents such as Ebola, Lassa, Pox, and severe acute respiratory syndrome viruses [117,118]. Results showed a lower detection rate for lineage 2 of WNV.

The second EQA of molecular methods showed an improved proficiency of laboratories compared to the first EQA performed in 2006. However, results suggest that detection of both WNV lineages is still problematic. Therefore, further proceedings for the detection of both lineages are needed, particularly for in-house assays [115].

For the first EQA of serological detection methods (2007), only 30% of participating laboratories passed the minimum requirements for successful performance. This result is mainly explained by the high rate of cross-reactivity with sera positive for related Abs, particularly those specific for YFV. Also the low sensitivity for IgM detection results in a risk of overlooking WNV acute infections. In agreement with a previous EQA study for the serological detection of dengue virus infection [119,120], there was no significant difference between the use of commercial or in-house assays.

The second EQA of serological detection methods was conducted in 2012 and involved 47 participants compared to 27 in 2007. Compared to the previous study, results still showed low specificity for the IgG detection methods demonstrating a high level of cross-reactivity with other flaviviruses. Likewise, low sensitivity of IgM detection was also observed indicating that there is still a need to improve WNV serological diagnostic tests. These studies demonstrate the importance of quality control measures in the detection of WNV infection. The results clearly indicate a need for laboratories to improve their tests: in particular by avoiding cross-reactivity with sera containing antibodies specific for heterologous flaviviruses and also by achieving lower detection limits in RT‑PCR tests for all WNV lineages. By organising such comparative testing of well-characterised samples, laboratories are given the opportunity to identify their weaknesses and to improve their methodologies, which should then be confirmed in subsequent studies.

## 5. Conclusions

The diagnosis of WNV infections should be primarily based on clinical evidence [121] and on laboratory data obtained from standardized and controlled procedures (see above). The laboratory requires minimal information (such as onset of symptoms, major clinical features and demographic information) to perform the adequate assays according to the clinical phase. Often the use of several assays is required for confirming the detection of WNV infection. In addition, the diversity and continuous evolution of circulating strains require regular revision of the molecular assays. Of utmost importance is the periodical participation of the diagnostic laboratories in the EQA studies in order to maintain and possibly improve their performance in detecting WNV infection.
